# The efficacy of diltiazem, glyceryl trinitrate, nifedipine, minoxidil, and lidocaine for the medical management of anal fissure: a systematic review and network meta-analysis of randomized controlled trials

**DOI:** 10.1097/JS9.0000000000002263

**Published:** 2025-01-30

**Authors:** Cong Wang, Jing Ni, Yi Xiong, Jinlan Chen, Binting Li, Li Xu

**Affiliations:** a The First Affiliated Hospital of Zhejiang Chinese Medical University (Zhejiang Provincial Hospital of Traditional Chinese Medicine), Hangzhou, China; b Graduate School, Zhejiang Chinese Medical University, Hangzhou, China; c Department of Anorectal Surgery, The First Affiliated Hospital of Zhejiang Chinese Medical University (Zhejiang Provincial Hospital of Traditional Chinese Medicine), Hangzhou, China

**Keywords:** anal fissure, network meta-analysis, non-surgical treatments, systematic review

## Abstract

**Purpose:**

Anal fissure (AF) is a common anorectal condition causing pain, bleeding, and other perianal discomfort. This study conducts a network meta-analysis (NMA) to compare the efficacy and side effect profiles of pharmacological treatments for AF, including diltiazem (DTZ), glyceryl trinitrate (GTN), nifedipine (ND), minoxidil (MD), and lidocaine (LC).

**Methods:**

Following the Preferred Reporting Items for Systematic Reviews and Meta-Analysis guidelines, a systematic review and NMA were performed. Randomized controlled trials (RCTs) comparing non-surgical treatments for AF were sourced from PubMed, Cochrane Library, Embase, and Medline. The primary outcome was AF healing, with secondary outcomes including recurrence rates, pain reduction (visual analog scale), and adverse effects. Statistical analysis utilized odds ratios and surface under the cumulative ranking values for treatment ranking.

**Results:**

Twenty-two RCTs involving a total of 1770 participants were included in the analysis. ND demonstrated the highest healing rate, followed by DTZ and MD. LC exhibited the lowest healing rate. DTZ had the lowest recurrence rate and was the most effective in pain reduction, whereas LC had the highest recurrence rate and was the least effective in alleviating pain. The incidence of adverse effects with MD was relatively low, second only to LC, while GTN had the highest rate of adverse effects.

**Conclusion:**

ND had the highest healing rate and should be considered as a first-line non-surgical treatment for AFs. Given the elevated incidence of adverse reactions associated with nitroglycerin, its use should be avoided in order to minimize the risk of significant toxicities and side effects. Additionally, because of its outstanding analgesic properties, DTZ is recommended as the preferred option for patients with heightened sensitivity to pain, but more studies are needed to evaluate its efficacy.

## Introduction

An anal fissure (AF) is a common benign anorectal disease defined as a longitudinal tear or defect in the skin of the anal canal, located distal to the dentate line, which negatively affects patients’ quality of life^[1,2]^. Debated though the exact etiology is, factors like traumatic anal sex, chronic constipation, hemorrhoidectomy, vaginal delivery, and chronic cough are associated with elevated internal anal sphincter (IAS) pressure^[3-6]^. Therefore, the primary treatment goal is to reduce sphincter resting pressure and improve blood flow. For acute fresh AF, the American Society of Colon and Rectal Surgeons recommends non-surgical treatment, particularly topical glyceryl trinitrate (GTN) and calcium channel blockers (CCBs), as first-line therapy (1B). Moreover, lateral internal sphincterotomy is advised for chronic AFs in patients unresponsive to medical therapy (1A)^[2,7,8]^. While surgery remains the gold standard for the treatment of chronic AF, medical therapy is still considered as first-line therapy in our society to reduce the workload on hospitals and keeping in view the financial constraints of the patient^[9]^.

**Highlights**

- This study is the first NMA to evaluate the efficacy of commonly used pharmacological treatments in clinical practice of AF.
- Nifedipine demonstrated the highest healing rate among treatments, establishing it as the preferred first-line therapy.
- Diltiazem emerged as the most effective agent for pain relief, making it an ideal option for patients with heightened sensitivity to pain.
- Glyceryl trinitrate, despite its effectiveness, is associated with significant adverse effects, emphasizing the need for cautious use.

Topical GTN for AF has been widely studied since the 1990s, with a healing rate of up to 80%, and the main side effect is related to headache^[10]^. CCBs, such as diltiazem (DTZ) and nifedipine (ND), effectively reduce anal resting pressure and show significantly better healing rates than placebo with minimal side effects.^[11-14]^. Lidocaine (LC), a local anesthetic, provides symptom relief, but studies show that GTN achieves earlier and higher healing rates with comparable recurrence^[15]^. Recently, topical minoxidil (MD) has emerged as a promising treatment, with studies reporting faster and superior healing compared to 0.2% GTN cream^[16,17]^.

An extensive review of previous literature shows that the efficacy of DTZ, GTN, ND, MD, and LC have been studied and validated independently many times. However, no studies were found that directly compare these five treatments for AF in any of the online databases searched. Therefore, the aim of our study is to use a network meta-analysis (NMA) to assess and compare the efficacy of these five treatments for AF. This approach allows for simultaneous comparison of multiple treatments and provides a relative ranking probability for each treatment. The findings of this study may help guide the selection of the most appropriate treatment for patients with AFs.

## Methods

The authors conducted a systematic review and NMA in accordance with the Preferred Reporting Items for Systematic Reviews and Meta-Analysis (PRISMA) guidelines, with extension for NMA. This work had been reported in line with PRISMA and Assessing the Methodological Quality of Systematic Reviews Guidelines^[18,19]^.

### Search strategy

A comprehensive systematic search of the following four databases was performed: PubMed, Cochrane Library, Embase, and Medline. The final search was conducted on 29 May 2024. Additionally, references from the included studies were reviewed for potentially relevant publications. The search strategy’s specifics can be found in Supplementary Table S1, Supplemental Digital Content, http://links.lww.com/JS9/D854.

### Inclusion and exclusion criteria

The inclusion criteria for our study encompassed: (1) randomized controlled trials (RCTs) comparing treatments for AF and (2) only studies reporting medical treatments were included. The exclusion criteria for the study included: (1) not written in English; (2) RCTs published before 1 January 1990; (3) studies reporting surgical treatments; and (4) studies reporting on Botox injected;

### Study selection and data collection

The trials for inclusion were identified by two authors through independent screening of titles and abstracts of records. Full texts were sought for any potentially relevant records by at least one author, and these were also independently screened for inclusion. Any disagreements were resolved through discussion among the two authors and with a senior author where necessary. The following data were independently extracted from each study by three authors: first author, year of publication, country, inclusion and exclusion criteria, sample size, participant characteristics, and the outcomes of interest. Any discrepancies were resolved by discussion, and a final decision was taken by an author.

### Outcomes of interest

The primary outcome was AF healing, defined by clinical examination of the patient at follow-up or by self-reported patient symptoms of absence of pain. Secondary outcomes included recurrence rate, adverse effects, and pain. Pain was scored on the visual analog scale (VAS).

### Bias assessment

The Cochrane Collaboration’s Risk of Bias tool 2.0 was used to assess the risk of bias of the included trials based on the following domains: randomization process, deviation from intended interventions, missing outcome data, measurement of the outcome, and selection of the reported result. For each of these risk domains, the studies were categorized as being at low, uncertain, or high risk of bias. The overall risk of bias of studies was calculated according to the algorithm’s overall judgment.

### Statistical analysis

The STATA software version 18.0, Review Manager 5.4, and GeMTC14.3 were used for the NMA. The healing rate, recurrence rate, adverse effects, and VAS score were compared between each treatment. The effect sizes of both dichotomous and continuous variables were estimated by the odds ratio (OR). Continuous data that were presented as the median and range or interquartile range (IQR) were converted to the mean and standard deviation (SD). If the SD was not reported, then it was calculated from the standard error, probability (P) value, confidence interval (CI), or IQR according to guidance from the Cochrane Handbook for Systematic Reviews of Interventions. Results of each outcome were illustrated in a league table, showing the OR (with 95% CI) for each treatment comparison, surface under the cumulative ranking curve (SUCRA), showing each treatment ranking with its respective ranking probability. The STATA 18.0 software was used to plot the reticular relationship graphs and calculate closed loops’ inconsistency factor (IF) by the 95% CI. It showed that the direct and indirect evidence were consistent if the 95% CI of IF contained ʻoʼ. Otherwise, there was a high possibility of inconsistency.

## Results

**Figure 1.**
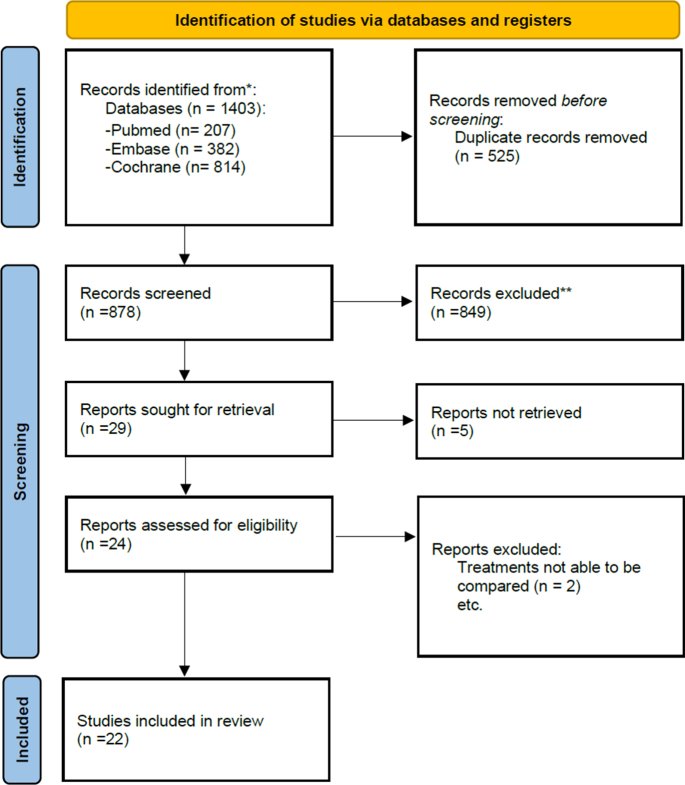
PRISMA flowchart of included studies.

A total of 878 records were screened for relevance using their title and/or abstract after removal of duplicates. Twenty-nine full-text articles were then assessed, and of these, 22 were included in this NMA. Figure 1 shows the PRISMA flowchart of the included studies. Table 1 displays a summary of the studies included in the NMA.

**Table 1:** Study characteristics

Author and year	Treatments	Total participants	Follow-up period (weeks)
Zahra, 2023	ND	53	1, 2, 3, 4, 5, 6, 7, 8, 24
	DTZ	50	
Hareth, 2022	DTZ	60	1, 2, 8, 24
	GTN	52	
Archana, 2020	ND	33	1, 2, 3, 4, 8, 24, 48
	DTZ	33	
	Lignocaine	34	
Ozan, 2020	GTN	50	3, 5, 12
	ND	50	
Emile, 2020	GTN	32	2, 4
	Minoxidil	30	
Alvandipour, 2017	DTZ	44	2, 4, 6, 8
	Minoxidil	44	
Suman, 2017	GTN	35	2, 4, 6, 8, 24
	ND	40	
Muhammad, 2017	GTN	92	4
	DTZ	92	
Amyn, 2014	DTZ	30	2
	GTN	30	
Shahram, 2012	DTZ	36	2, 4, 8
	GTN	25	
Hanumanthappa, 2012	DTZ	100	2, 4, 6
	Lignocaine	100	
Muazez, 2012	GTN	28	1, 2, 3, 4, 6, 8, 12, 16, 20, 24, 28, 32, 36, 40, 44, 48
	DTZ	28	
	Lignocaine	28	
Masood, 2009	GTN	35	2, 4, 6, 8
	DTZ	38	
Sanei, 2009	DTZ	51	2, 4, 6, 8, 10, 12
	GTN	51	
Fazila, 2008	DTZ	47	2, 4, 6, 8
	GTN	50	
Ahmad, 2007	GTN	25	1, 2, 8, 24
	Lignocaine	25	
Shrivastava, 2007	DTZ	30	2, 4, 6, 12
	GTN	30	
Mustafa, 2006	GTN	10	2, 4, 6, 8
	ND	10	
Muthukumarassamy, 2005	Minoxidil	34	1, 2, 3, 4, 5, 6
	Lignocaine	27	
Tiberiu, 2003	ND	26	2, 4, 6, 8, 24
	GTN	26	
Bielecki, 2002	DTZ	22	2, 8
	GTN	21	
Kocher, 2002	GTN	29	3, 6, 9, 12
	DTZ	31	

DTZ, diltiazem; GTN, glyceryl trinitrate; ND, nifedipine.

### Risk of bias analysis

**Figure 2.**
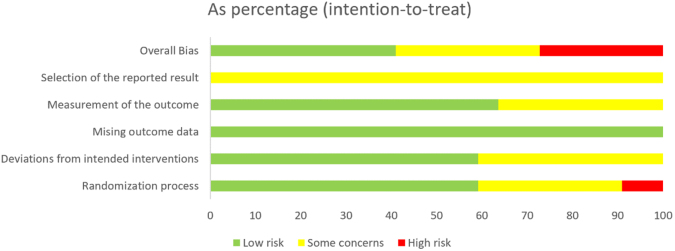
Risk of bias according to Cochrane risk of bias tool 2.0.

The risk of bias in the included trials is summarized in Figure 2 and described for each study in Appendix S1, Supplemental Digital Content, http://links.lww.com/JS9/D855. There were six (27.3%) RCTs judged as being at ‘high’ risk of bias.

#### Network meta-analysis of healing rate

**Figure 3.**
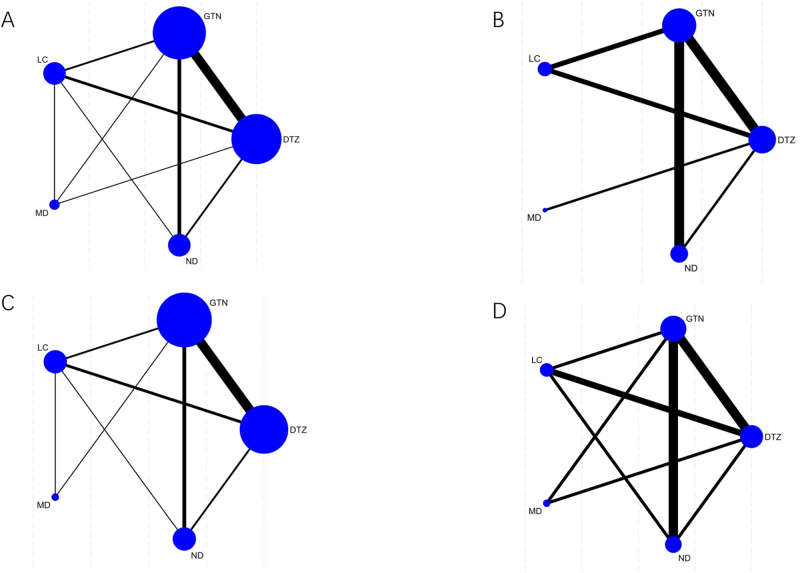
Reticulated evidence diagrams of (A) healing rate, (B) recurrence rate, (C) decrease of VAS score, and (D) adverse effects rate. VAS indicates visual analog scale.

**Figure 4.**
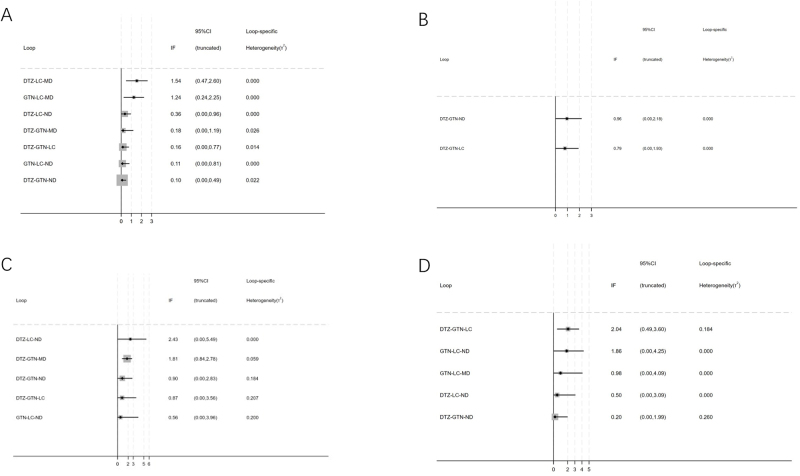
Inconsistency test of (A) healing rate, (B) recurrence rate, (C) decrease of VAS score, and (D) adverse effects rate. VAS indicates visual analog scale.

Twenty-one studies reported the healing rate related to five methods. The result of the cure rate contained seven closed loops, including DTZ-LC-MD, GTN-LC-MD, DTZ-LC-ND, DTZ-GTN-MD, DTZ-GTN-LC, GTN-LC-ND, and DTZ-GTN-ND (Fig. 3A). The 95% CI of IF in the five of seven closed loops contained ʻ0ʼ, including DTZ-LC-ND, DTZ-GTN-MD, DTZ-GTN-LC, GTN-LC-ND, and DTZ-GTN-ND, demonstrating no significant inconsistency between the direct and indirect evidence in these loops (Fig. 4A). The other two instances of inconsistency may be due to loop inconsistency arising from significant heterogeneity between the studies. It is likely due to studies of different comparisons being undertaken in different periods, settings, or contexts, and these differences are associated with the magnitude of the treatment effect. According to the NMA results, significant results are in bold (Table 2).

**Table 2:** Network meta-analysis results of healing rate (OR, 95% CI)

Network meta-analysis results of healing rate (OR, 95% CI)				
GTN	**2.09 (1.36, 3.22)**	**3.81 (1.92, 7.55)**	1.75 (0.70, 4.36)	**0.30 (0.15, 0.62)**
**0.48 (0.31, 0.74)**	DTZ	1.82 (0.88, 3.76)	0.84 (0.34, 2.06)	**0.15 (0.07, 0.29)**
**0.26 (0.13, 0.52)**	0.55 (0.27, 1.13)	ND	0.46 (0.15, 1.37)	**0.08 (0.03, 0.20)**
0.57 (0.23, 1.43)	1.20 (0.49, 2.94)	2.18 (0.73, 6.52)	MD	**0.17 (0.07, 0.46)**
**3.29 (1.62, 6.69)**	**6.87 (3.47, 13.60)**	**12.52 (5.06, 31.00)**	**5.75 (2.18, 15.12)**	LC

CI, confidence interval; DTZ, diltiazem; GTN, glyceryl trinitrate; LC, lidocaine; MD, minoxidil; ND, nifedipine; OR, odds ratio.

Significant results are in bold.

**Figure 5.**
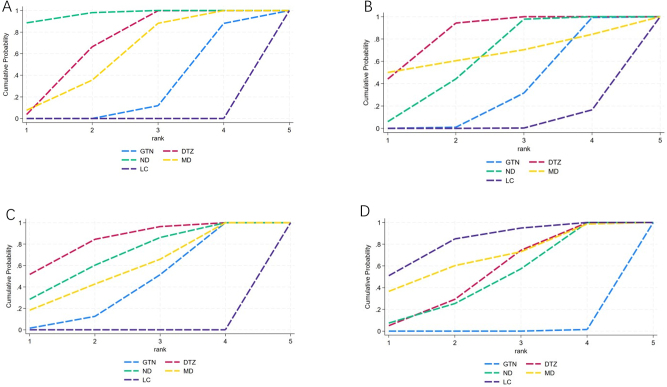
SUCRA plot of (A) healing rate, (B) recurrence rate, (C) decrease of VAS score, and (D) adverse effects rate. VAS indicates visual analog scale.

Twenty-one studies compared five different treatments across 1770 participants. There were 10 unique pairwise comparisons. Overall, GTN, DTZ, ND, and MD had significantly greater odds of healing compared to LC. ND did not result in significantly greater odds of healing compared to DTZ (OR 1.82, 0.88–3.76) and MD (OR 2.18, 0.73–6.52) but had higher odds than LC (OR 12.52, 5.06–31.00) and GTN (OR 3.81, 1.92–7.55). MD did not have greater odds of healing compared to GTN (OR 1.75, 0.70–4.36). DTZ performed significantly better than GTN (OR 2.09, 1.36–3.22), but there was no significant difference between DTZ and ND (OR 1.82, 0.88–3.76). SUCRA values, from highest to lowest, were 96.6 for ND, 67.5 for DTZ, 57.8 for MD, 28.0 for GTN, and 0 for LC. The SUCRA plot is displayed in Figure 5A.

#### Network meta-analysis of recurrence rate

Twelve studies reported the recurrence rate related to five methods. The result of the recurrence rate contained two closed loops: DTZ-GTN-ND and DTZ-GTN-LC (Fig. 3B). The 95% CI of IF in the two closed loops contained ʻ0ʼ, which showed no significant inconsistency between the direct and indirect evidence (Fig. 4B). According to the NMA results, significant results are in bold (Table 3).

**Table 3:** Network meta-analysis results of recurrence rate (OR, 95% CI)

Network meta-analysis results of recurrence rate (OR, 95% CI)				
GTN	**0.33 (0.17, 0.64)**	0.55 (0.29, 1.04)	0.33 (0.01, 18.68)	**2.68 (1.18, 6.08)**
**3.02 (1.55, 5.85)**	DTZ	1.67 (0.74, 3.77)	1.00 (0.02, 53.34)	**8.07 (3.61, 18.06)**
1.80 (0.96, 3.40)	0.60 (0.27, 1.35)	ND	0.60 (0.01, 34.66)	**4.83 (1.78, 13.13)**
3.02 (0.05, 169.98)	1.00 (0.02, 53.34)	1.67 (0.03, 96.80)	MD	8.07 (0.14, 466.78)
**0.37 (0.16, 0.85)**	**0.12 (0.06, 0.28)**	**0.21 (0.08, 0.56)**	0.12 (0.00, 7.16)	LC

CI, confidence interval; DTZ, diltiazem; GTN, glyceryl trinitrate; LC, lidocaine; MD, minoxidil; ND, nifedipine; OR, odds ratio.

Significant results are in bold.

Twelve studies compared five different treatments across 876 participants. There were 10 unique pairwise comparisons. GTN, DTZ, and ND had significantly lower odds of recurrence compared to LC. ND did not result in lower recurrence rates compared to GTN (OR 0.55, 0.29–1.04). DTZ demonstrated a significantly lower odds of recurrence compared to GTN (OR 0.33, 0.17–0.64), but there was no significant difference between DTZ and ND (OR 0.60, 0.27–1.35). MD did not result in lower recurrence rate compared to ND (OR 0.60, 0.01–34.66) and GTN (OR 0.33, 0.01–18.68). SUCRA values, from highest to lowest, were 84.9 for DTZ, 66.4 for MD, 61.7 for ND, 32.9 for GTN, and 4.1 for LC. The SUCRA plot is displayed in Figure 5B.

#### Network meta-analysis of decrease of VAS score

Eleven studies reported the recurrence rate relating to five methods. The result of the decrease of VAS score contained five closed loops: DTZ-LC-ND, DTZ-GTN-MD, DTZ-GTN-ND, DTZ-GTN-LC, and GTN-LC-ND (Fig. 3C). The 95% CI of IF in the four of five closed loops contained ʻ0ʼ, which showed no significant inconsistency between the direct and indirect evidence (Fig. 4C). According to the NMA results, significant results are in bold (Table 4).

**Table 4:** Network meta-analysis results of decrease of VAS score (OR, 95% CI)

Network meta-analysis results of decrease of VAS score (OR, 95% CI)				
GTN	1.70 (0.80, 3.61)	1.39 (0.66, 2.93)	1.18 (0.41, 3.36)	**0.06 (0.02, 0.19)**
0.59 (0.28, 1.25)	DTZ	0.82 (0.31, 2.16)	0.69 (0.24, 1.99)	**0.03 (0.01, 0.11)**
0.72 (0.34, 1.52)	1.22 (0.46, 3.23)	ND	0.85 (0.24, 2.96)	**0.04 (0.01, 0.15)**
0.85 (0.30, 2.42)	1.44 (0.50, 4.12)	1.18 (0.34, 4.11)	MD	**0.05 (0.01, 0.22)**
**18.14 (5.25, 62.74)**	**30.81 (8.81, 107.79)**	**25.19 (6.74, 94.17)**	**21.39 (4.58, 99.92)**	LC

CI, confidence interval; DTZ, diltiazem; GTN, glyceryl trinitrate; LC, lidocaine; MD, minoxidil; ND, nifedipine; OR, odds ratio; VAS, visual analog scale.

Significant results are in bold.

Eleven studies compared five different treatments across 873 participants. There were 10 unique pairwise comparisons. Overall, GTN, DTZ, ND, and MD had significantly greater odds of decrease of VAS score compared to LC. DTZ did not result in a significantly greater odds of decrease of VAS score compared to GTN (OR 1.70, 0.80–3.61), ND (OR 1.22, 0.46–3.23), and MD (OR 1.44, 0.50–4.12). ND did not have greater odds of decrease of VAS compared to GTN (OR 1.39, 0.66–2.93) and MD (OR 1.18, 0.34–4.11). MD did not have greater odds of decrease of VAS compared to GTN (OR 1.18, 0.41–3.36). SUCRA values, from highest to lowest, were 83.1 for DTZ, 68.8 for ND, 56.7 for MD, 41.3 for GTN, and 0 for LC. The SUCRA plot is displayed in Figure 5C.

#### Network meta-analysis of adverse effects rate

Twenty studies reported the recurrence rate related to five methods. The result of the recurrence rate contained five closed loops: DTZ-GTN-LC, GTN-LC-ND, GTN-LC-MD, DTZ-LC-ND, and DTZ-GTN-ND (Fig. 3D). The 95% CI of IF in the four of five closed loops contained ʻ0ʼ, which showed no significant inconsistency between the direct and indirect evidence (Fig. 4D). According to the NMA results, significant results are in bold (Table 5).

**Table 5:** Network meta-analysis results of adverse effects rate (OR, 95% CI)

Network meta-analysis results of adverse effects rate (OR, 95% CI)				
GTN	**0.13 (0.06, 0.28)**	**0.15 (0.05, 0.46)**	**0.09 (0.01, 0.81)**	**0.06 (0.02, 0.25)**
**7.64 (3.57, 16.32)**	DTZ	1.14 (0.34, 3.77)	0.66 (0.07, 6.76)	0.48 (0.12, 1.86)
**6.70 (2.16, 20.74)**	0.88 (0.27, 2.90)	ND	0.58 (0.05, 6.97)	0.42 (0.08, 2.23)
**11.52 (1.23, 107.49)**	1.51 (0.15, 15.38)	1.72 (0.14, 20.61)	MD	0.72 (0.06, 8.18)
**16.03 (4.05, 63.34)**	2.10 (0.54, 8.19)	2.39 (0.45, 12.74)	1.39 (0.12, 15.84)	LC

CI, confidence interval; DTZ, diltiazem; GTN, glyceryl trinitrate; LC, lidocaine; MD, minoxidil; ND, nifedipine; OR, odds ratio.

Significant results are in bold.

Twenty studies compared five different treatments across 1575 participants. There were 10 unique pairwise comparisons. DTZ, ND, MD, and LC had significantly lower odds of adverse effects rate compared to GTN. DTZ did not result in lower adverse effects rate compared to ND (OR 0.88, 0.27–2.90). MD did not result in lower adverse effects rate compared to ND (OR 0.58, 0.05–6.97) and DTZ (OR 0.66, 0.07–6.67). SUCRA values, from highest to lowest, were 82.7 for LC, 67.1 for MD, 52.2 for DTZ, 47.6 for ND, and 0.4 for GTN. The SUCRA plot is displayed in Figure 5D.

## Discussion

An AF is one of the most frequently encountered anorectal disorders in clinical practice. It is widely acknowledged that sphincter hypertonicity resulting from traumatic injury may result in elevated anal canal pressure, subsequently leading to localized ischemia of the anal mucosa^[20]^. GTN is the most commonly employed chemical agent in the treatment of AFs, as it effectively reduces anal sphincter tonicity through its non-adrenergic mechanisms^[21]^. The transport of calcium through L-type calcium channels is crucial for maintaining the tone of the IAS. By preventing the intracellular access of calcium, CCBs like ND and DTZ interrupt the fissure cascade by decreasing spontaneous sphincter activity^[22]^. LC provides analgesic effects by blocking pain signal transmission from afferent nerve fibers to the central nervous system. This mechanism is primarily due to its affinity for binding to voltage-gated sodium channels, which reversibly inhibits neuronal action potentials and prevents nerve impulse conduction^[23]^. MD induces hyperpolarization of smooth muscle through the opening of potassium channels and the closure of voltage-operated calcium channels, which results in the relaxation of blood vessel smooth muscles and the IAS^[24]^.

As mentioned previously, it is not difficult to conclude from previous literature that DTZ, ND, MD, GTN, and LC have satisfying efficacy in the treatment of AF. However, according to the results of this article, we found that these five drugs have different performances in drug cure rate, recurrence rate, pain relief, and adverse reaction rate.

In terms of cure rate, we found that all interventions demonstrated superior effectiveness compared to LC. ND showed the most significant impact on promoting AF healing, followed by DTZ and then MD, with nitroglycerin being the least effective. Previous research comparing GTN with DTZ and ND has also shown that GTN has a lower cure rate than DTZ and ND. For instance, Hassan *et al*.^[25]^ conducted a prospective randomized trial with 112 patients with AFs over 1.5 years, finding complete fissure healing in 83.3% of the DTZ group and 76% of the GTN group. Similarly, Shrestha *et al*.^[26]^ studied 90 patients randomly assigned to GTN or ND groups, with 45 patients each, and reported a healing rate of 82.5% in the ND group compared to 60% in the GTN group. Among the above five drugs, ND and DTZ are both classified as CCBs. Apart from decreasing spontaneous sphincter activity, CCBs promote AF healing through multiple mechanisms. CCBs also exhibit antioxidant properties that protect tissues from oxidative damage and increase nitric oxide (NO) production, which is essential for angiogenesis and the proliferation of key cells involved in healing, such as fibroblasts, epithelial cells, and keratinocytes. In the meantime, they can enhance collagen accumulation and fibroblast proliferation, critical components of tissue repair. Moreover, CCBs may help alleviate chronic inflammation due to their higher vaso-selectivity for peripheral vascular smooth muscles and their ability to prevent increases in vascular permeability. Their vasodilatory effects improve blood flow to the wound area, providing vital nutrients and oxygen while stimulating the production of growth factors. Furthermore, CCBs regulate the activity of tissue collagenases, such as matrix metalloproteinases, facilitating the remodeling of the extracellular matrix. Collectively, these actions contribute to an accelerated and more effective wound-healing process^[27,28]^. So, both ND and DTZ showed a better cure rate than other drugs. However, there are still some differences between ND and DTZ. They consist of a heterogeneous array of compounds with distinct chemical structures, resulting in varying potencies for calcium channel blockade. DTZ can inhibit calcium influx in both cardiac and vascular smooth muscle cells, whereas ND exhibits a greater potency in promoting the relaxation of peripheral smooth muscle cells^[13]^. This study found that ND is superior to DTZ in promoting the healing of AFs. This advantage is likely due to the fact that non-dihydropyridine CCBs, such as DTZ, are less selective than dihydropyridine CCBs, such as ND, in peripheral blood vessels and do not induce reflex sympathetic nerve activation, thereby reducing anal pressure^[29,30]^.

MD exhibits a selective vasodilatory effect, primarily affecting the coronary arteries, gastrointestinal vessels, and cerebral vessels. Its vasodilatory action on renal and cutaneous vasculature is inferior to that of CCBs. This may elucidate why MD demonstrates lower efficacy in terms of treatment success compared to ND and DTZ^[16,31]^. GTN is able to dilate perianal blood vessels to help treat AF, whose function is so single that its cure rate is not as high as that of CCBs. Also, due to its significant side effects, a considerable number of cases involving GTN were withdrawn from the study prior to its completion, which may contribute to its relatively low healing rate. LC, as a sodium channel blocker that acts on nerve cells, provides only analgesic effects without the ability to reduce resting pressure in the anal sphincter or improve blood flow. This may contribute to its relatively poor performance in terms of healing rates.

Following pharmacological treatment, anal canal pressure remains at levels comparable to those before therapy, leading to a notably high recurrence rate^[20]^. In terms of recurrence rates, our research indicates that DTZ exhibits the lowest recurrence rate among various interventions, followed by MD, GTN, and, lastly, LC, which ranked the lowest. The mechanism underlying the impact of DTZ on the recurrence of AFs remains unclear; potential factors contributing to its efficacy may include DTZ’s prolonged duration of action and fewer side effects compared to ND. Cevik *et al*.^[32]^ reported a recurrence rate of 11.1% in the DTZ group, compared to 37% for the GTN group and 57% for the LC group. Meanwhile, in the study by Momayez Sanat *et al*.^[33]^, the ND group exhibited a recurrence rate of 16.3% after 3 months, whereas the DTZ group had a rate of 24.1%. This study primarily investigates the recurrence rates after pharmacological treatment of AFs with ND, DTZ, MD, GTN, and LC; however, the results may be subject to influences from follow-up duration, the nature and severity of the individual’s AFs, comorbidities, lifestyle and dietary habits, local care practices, psychological factors, and compliance with medical advice^[34]^. Due to the limitations of sample size or the potential for excessive data variability, the advantage of DTZ in reducing the recurrence rate of AFs warrants validation in future studies.

Regarding analgesic effects, our research found that, compared to LC, all other interventions demonstrated superior analgesic effects. Currently, no studies have proven that DTZ possesses a unique advantage in pain relief. According to the SUCRA values, DTZ ranked highest in analgesic effect, followed by ND, then MD, GTN, and, lastly, LC. This may be attributed to DTZ’s superior performance in alleviating sympathetic nervous system overactivity compared to other interventions, as increased sympathetic vascular tone and cardiac sympathetic dominance may play a significant role in patients with VSA^[35]^. Meanwhile, in this study, the primary criterion for evaluating analgesic effects in the included literature was the VAS score. VAS is a commonly used subjective assessment tool widely applied to evaluate patients’ pain levels, symptom severity, and other subjective experiences. It typically manifests as a 10-cm or 100-mm line with two end points indicating extreme states, such as “no pain” and “extreme pain.” Patients indicate their feelings by marking this line, and researchers can quantify the score by measuring the distance from one end point to the mark^[36]^. Despite its ease of comprehension and use, leading to its application in numerous clinical studies, VAS scores are highly dependent on patients’ self-assessments, which may be influenced by individual emotions, cultural backgrounds, and other factors, potentially resulting in inconsistent scores. VAS assumes a linear perception of pain or symptoms; however, this perception can often be nonlinear, especially during fluctuations in emotional states. These factors may also impact the findings of this study.

On the aspect of the rate of adverse effects, Hassan *et al*.^[25]^ reported that among 52 patients in the GTN group, 30 (57.6%) experienced headache, 12 (23%) experienced pruritus, and 3 (5.7%) experienced hypotension. Ala *et al*.^[37]^ found that all 25 patients in the GTN group reported headache, with 12 cases of constipation and 10 cases of pruritus. In contrast, only two of 36 patients in the DTZ group experienced constipation, and three reported pruritus. Shrestha *et al*.^[26]^ noted that among 43 patients in the ND group, three experienced headaches, while seven out of 42 patients in the GTN group also reported headache. Our study found that the adverse reactions associated with these medications primarily included headache, hypotension, pruritus, gastrointestinal disturbances, vertigo, incontinence, and constipation, encompassing nearly all adverse effects reported in previous research. Among these adverse effects, headache is the most common adverse effect associated with GTN, which may be attributed to its role as a NO donor. The increased incidence of migraines can be linked to the enhanced enzymatic activity and/or affinity resulting from the NO-triggered cascade of reactions^[38]^. This finding is consistent with our results, indicating that GTN has a comparatively higher incidence of adverse reactions compared to other interventions. In terms of SUCRA values, LC ranked highest, followed by MD, then DTZ, ND, and, finally, GTN. As a commonly used local anesthetic, LC exhibits minimal side effects in the treatment of AFs. The primary adverse reaction associated with MD is pruritus, which could impact patient adherence^[24]^. This may be related to differences in concentration; the 5% MD formulation contains a higher concentration of propylene glycol (50%) compared to the 2% formulation (30%) and is associated with an increased incidence of itching, erythema, and dryness. Propylene glycol may contribute to the development of allergic contact dermatitis in response to MD solutions^[39]^.

This study is the first NMA to evaluate the efficacy of commonly used pharmacological treatments in clinical practice of AF. Nevertheless, certain details remain unclear and debatable based on the existing literature. For example, the pairwise comparisons between DTZ and ND showed no statistical significance across all outcome measures, and there are also other “no-statistical-significances” between different comparisons. Given the lack of relevant studies and insufficient sample sizes, further research is needed in the future to compare these five drugs and verify the results of our study. At the same time, follow-up durations for the outcomes of interest may have varied between studies, contributing to heterogeneity in our findings. Furthermore, certain treatments such as MD and LC were estimated indirectly based on a limited number of studies, leading to reduced precision in the findings. Therefore, the duration of the study and the number of subjects must be taken into consideration in future investigations.

## Conclusion

ND had the highest healing rate and should be considered as a first-line non-surgical treatment for AFs. Given the elevated incidence of adverse reactions associated with nitroglycerin, its use should be avoided in order to minimize the risk of significant toxicities and side effects. Additionally, because of its outstanding analgesic properties, DTZ is recommended as the preferred option for patients with heightened sensitivity to pain, but more studies are needed to evaluate its efficacy.

## Data Availability

The following data are publicly available. Further inquiries can be directed to the corresponding author, including the template data collection forms, data extracted from included studies, data used for all analyses, the analytic code, and any other materials used in the review.

## Research registration unique identifying number (UIN)

National Institute for Health and Care Research, CRD42024582992.
